# Antagonizing *cis-*regulatory elements of a conserved flowering gene mediate developmental robustness

**DOI:** 10.1073/pnas.2421990122

**Published:** 2025-02-18

**Authors:** Amy Lanctot, Anat Hendelman, Peter Udilovich, Gina M. Robitaille, Zachary B. Lippman

**Affiliations:** ^a^HHMI, Cold Spring Harbor Laboratory, Cold Spring Harbor, NY 11724; ^b^Plant Biology, Cold Spring Harbor Laboratory, Cold Spring Harbor, NY 11724

**Keywords:** *cis*-regulation, flower development, conserved noncoding sequence, canalization

## Abstract

Developmental transitions require precise temporal and spatial control of gene expression. In plants, such regulation is critical for flower formation, which involves the progressive maturation of stem cell populations within shoot meristems to floral meristems, followed by rapid sequential differentiation into floral organs. Across plant taxa, these transitions are orchestrated by the F-box transcriptional cofactor gene *UNUSUAL FLORAL ORGANS* (*UFO*). The conserved and pleiotropic functions of *UFO* offer a useful framework for investigating how evolutionary processes have shaped the intricate *cis-*regulation of key developmental genes. By pinpointing a conserved promoter sequence in an accessible chromatin region of the tomato ortholog of *UFO*, we engineered in vivo a series of *cis-*regulatory alleles that caused both loss- and gain-of-function floral defects. These mutant phenotypes were linked to disruptions in predicted transcription factor binding sites for known transcriptional activators and repressors. Allelic combinations revealed dosage-dependent interactions between opposing alleles, influencing the penetrance and expressivity of gain-of-function phenotypes. These phenotypic differences support that robustness in tomato flower development requires precise temporal control of *UFO* expression dosage. Bridging our analysis to *Arabidopsis*, we found that although homologous sequences to the tomato regulatory region are dispersed within the *UFO* promoter, they maintain similar control over floral development. However, phenotypes from disrupting these sequences differ due to the differing expression patterns of *UFO*. Our study underscores the complex *cis-*regulatory control of dynamic developmental genes and demonstrates that critical short stretches of regulatory sequences that recruit both activating and repressing machinery are conserved to maintain developmental robustness.

## Significance

Developmental processes are regulated by transcriptional networks requiring remarkable temporal and spatial control. Much of this regulation is mediated by noncoding sequence in the promoters of core developmental genes. In flowering plants, developmental transitions crucial for flower formation are controlled in large part by the conserved transcriptional regulator UNUSUAL FLORAL ORGANS (UFO). Focusing on homology in noncoding sequences, we identify deeply conserved sequences within the *UFO* promoter in two distally related flowering plants, tomato and *Arabidopsis*. CRISPR mutagenesis of these sequences resulted in opposing effects on flower formation, suggesting that such functionally dense sequences—likely common in intricately regulated developmental genes—are hubs of *cis-*regulatory activity that recruit both activating and repressing trans-acting factors.

*Cis-*regulatory control of developmental transitions has long been a topic of interest to geneticists—given the broad consistency of somatic genomes across cell types, temporal and spatial regulation of gene expression is the main mechanism by which the functional differentiation of cells that underpins development is achieved (1). Due to the complexity of gene expression regulation, parsing apart these *cis-*regulatory control nodes has been challenging. While the genomics era has ushered in a host of new strategies to assess transcription, the extreme context dependence of *cis-*regulation and the highly interconnected genetic interactions that regulate developmental transitions mean that even extensive global analyses of gene expression cannot bridge the gap to phenotype (2). To determine *cis-*regulatory functionality, a combination of genomic contextual data, including chromatin accessibility, transcription factor binding sites, and *cis*–trans interactions, can allow for more precision in our predictions (3).

Conservation across broad evolutionary distances can indicate that genomic sequence is under purifying selective pressure and cannot be mutated away without impeding function and phenotype (4). This has been a guiding principle of molecular evolutionary approaches, which focus on coding sequence conservation as the ratio of synonymous to nonsynonymous mutations can quantify selective pressures. Conserved noncoding sequences (CNSs) can also indicate functionality, and in animal systems, deeply CNSs are essential for organogenesis and body plan organization (5, 6). An influx of high-quality annotated genomes has made identification of CNSs across plants feasible (7). These sequences add another informative layer of genomic contextual knowledge to strategies that aim to predict and study *cis-*regulatory functionality.

Regulatory genes that exhibit pleiotropic activity during development are particularly promising candidates to address the still poorly understood relationships between noncoding sequence function and phenotype, as extreme precision in the expression patterns of these genes is indispensable for development (8). Consequently, querying *cis-*regulatory control of these fate drivers can dissect the extent to which these nodes are buffered and how changes in their expression affect the phenotypes they control (9). The regulatory sequences of such genes can impact both penetrance and expressivity of developmental phenotypes, as fine-tuning of their spatial and temporal expression patterns can lead to phenotypic changes of varying severities (10). Penetrance is the genetic concept that a change in phenotype does not manifest in all individuals carrying a particular mutation, both at the organ and organism scale, and expressivity is the related concept that the degree of a phenotype can differ between individuals (11). Despite historical descriptive work on these concepts, the molecular mechanisms behind incomplete penetrance and variable expressivity are poorly understood.

An essential developmental regulator in plants is *UNUSUAL FLORAL ORGANS* (*UFO*), whose pleiotropic roles in flowering and flower formation require tight temporal and spatial control of its expression and multilevel function. *UFO* was first identified in *Arabidopsis*, where null mutants show increased inflorescence branches due to the conversion of floral meristems into secondary shoots (12). Mutants develop flowers that lack petals and stamens, and different mutant alleles show variation in the expressivity of this phenotype, with some alleles causing complete petal loss in all flowers and others showing reduced petal number and homeotic conversions (12). Overexpression of *UFO* increases petal number and flower size (13). These phenotypes align with *UFO* expression in the meristem, as *UFO* is first expressed in the transitional meristem that precedes flower formation, and its expression is then spatially restricted to the inner whorls of the floral meristem, promoting petal and stamen development (14). These dual roles of UFO during meristem maturation and floral organ specification are reflected by the gene regulatory networks in which UFO is embedded during these developmental stages (15). During vegetative development of the shoot inflorescence meristem, expression of *LEAFY* (*LFY)*, a critical driver of flower formation and the DNA-binding transcription factor partner of *UFO*, is repressed by the antiflorigen *TERMINATING FLOWER 1* (*TFL1*) and the paralogous *CENTRORADIALIS* (*CEN*) genes (16). Meristem maturation promotes *LFY* expression, and it together with UFO activates the expression of essential flowering regulators, such as *APETALA1 (AP1)* (17). Once the floral transition has occurred, UFO and LFY promote expression of B-class MADS-box transcription factors, particularly *APETALA3* (*AP3*) to drive petal and stamen development. (18).

*UFO* presents a platform in which to study *cis-*regulatory function across evolution, as while its protein sequence and function is broadly conserved across flowering plants, its expression is not. In the Solanaceae, whose primary models for flowering are petunia and tomato, expression of *UFO* orthologs coincides with the floral transition and drives floral identity, whereas in the Brassicaceae *UFO* is also expressed in vegetative tissue (19). Disruption of *UFO* function from classical coding mutations, which impacts all functions in time, space, and level, consequently has more severe phenotypic consequences in Solanaceae species. For example, unlike *Arabidopsis* null *UFO* mutants, null mutants of the tomato *UFO* ortholog *ANANTHA* (*AN*) fail to make flowers, and instead indefinitely iterate inflorescence meristems and branches (20). Furthermore, transgenic overexpression of *UFO* in the Solanaceae species tobacco (13) and petunia (19) causes extremely early flowering. In tomato, precocious expression of *AN* results in a rapid transition to reproductive growth and single-flower inflorescences with large, leaf-like sepals. These phenotypes occur both in transgenic plants where *AN* is driven under the promoters of genes expressed earlier in meristematic maturation and in null mutants of an upstream repressor, *TERMINATING FLOWER* (*TMF*) (21). These strong opposing phenotypes from loss and gain of function imply that *AN* is under tight temporal and spatial control. Consequently, *AN cis-*regulation must have evolved to switch between activating and repressing regimes quickly as floral identity is established.

To dissect *UFO cis-*regulation, we used CRISPR to mutate CNSs in the *UFO* and *AN* promoters in their respective species. Perturbation of CNSs within a region of open chromatin in the tomato *AN* promoter strongly affected flowering. Distinct alleles resulted in loss- and gain-of-function mutant phenotypes, suggesting that this region is a hotspot of opposing *cis-*regulatory activity. Perturbation of CNSs in the *Arabidopsis UFO* promoter also affected flower development, with distinct CNSs again giving rise to both loss- and gain-of-function mutants. Our study showcases that targeting CNSs can generate allelic diversity revealing functionally dense *cis*-regulatory sequences that modulate penetrance and expressivity of phenotypes essential for both organism fitness and targeted developmental engineering.

## Results

### A Region of Open Chromatin and Conserved Sequence Is a “Hotspot” of *AN cis-*Regulation.

**Fig. 1.**
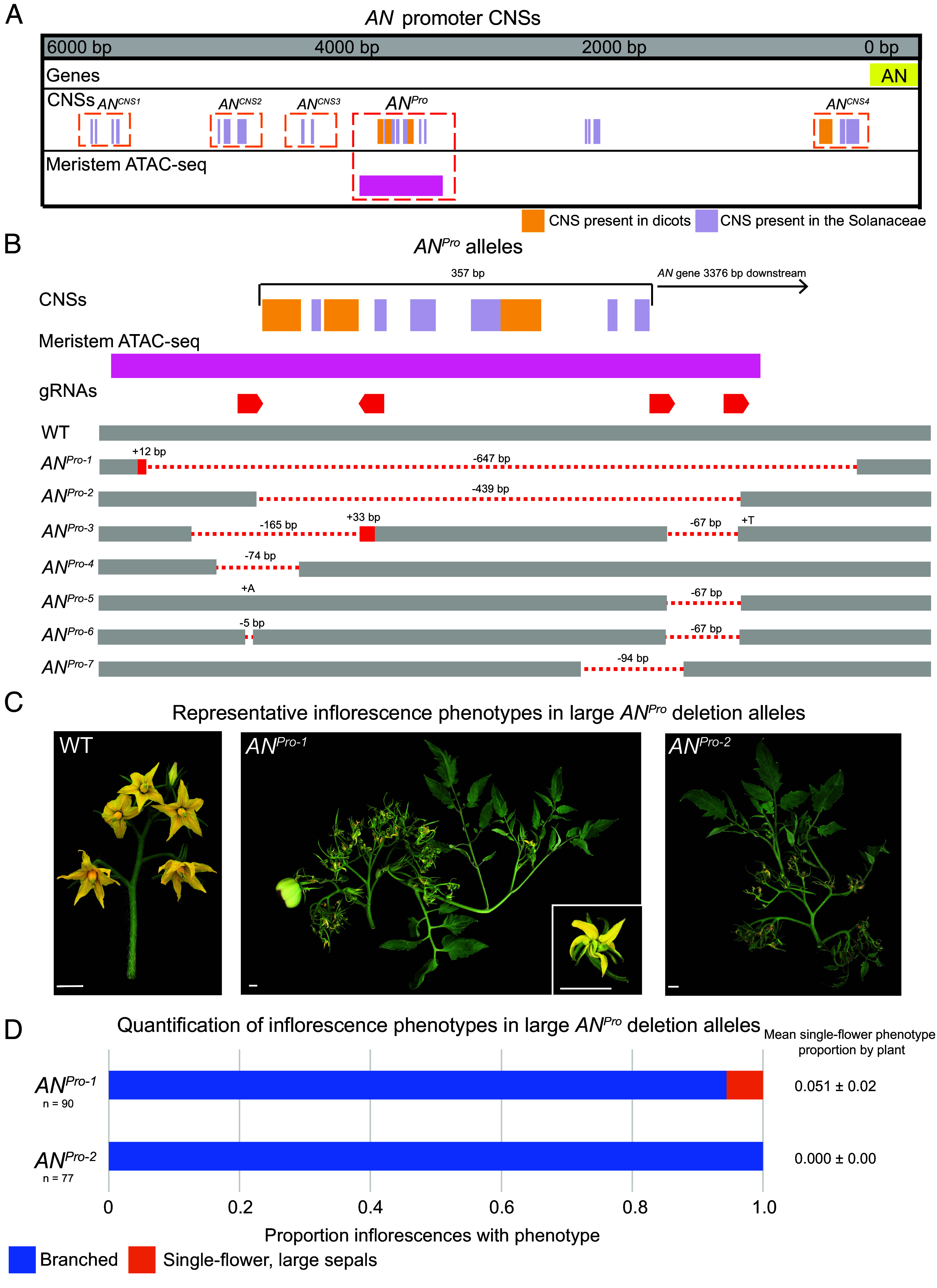
A conserved accessible region of the *AN* promoter is a hotspot for *cis*-regulatory function. (*A*) Visualization of CNSs within the *AN* promoter sequence. The *AN* promoter is defined as the ~ 6 kB between the *AN* coding sequence and the proximal upstream gene. CNSs are color-coded by their level of conservation and meristem ATAC-sequencing peaks are depicted. (*B*) Visualization of *AN^Pro^* allelic series. Alleles are ordered by the size and location of the deletion. (*C*) Representative inflorescences of WT, *AN^Pro-1^*, and *AN^Pro-2^* plants. The inset shows close-up of malformed flower formed in the branched phenotype. (Scale bar, 1 cm.) (*D*) Quantification of phenotypic frequency in *AN^Pro-1^* and *AN^Pro-2^* plants. The frequency of the single-flower phenotype per plant is given as a mean and SE.

To determine *AN cis-*regulatory sequence functionality, we leveraged our gene-centric ortholog-based alignment approach, Conservatory (7), to identify CNSs in the *AN* promoter. Conservatory categorizes CNSs by degree of conservation, i.e. the phylogenetic status of the species where conservation in orthologous *cis-*regulatory sequence can be identified. The majority of CNSs in the *AN* promoter are conserved across Solanaceae species, but four CNSs are also conserved to other dicot plant families (Fig. 1*A*). We used CRISPR-Cas9 genome editing to perturb five large stretches of the *AN* promoter that contained CNSs, targeting each region separately (Fig. 1*A*). For four of these regions, termed *AN^CNS1-4^* (*SI Appendix*, Fig. S1), we did not observe any changes in plant growth, inflorescence patterning, or flower development. This lack of phenotype could be due to inadequate allelic diversity, as limitations and stochasticity in CRISPR guide design and function does not allow for the complete loss of CNSs in these regions. Alternatively, these CNSs may act redundantly with other CNSs or nonconserved regions of the *AN* promoter, as additive and synergistic interactions among *cis*-elements can generate higher-order interactions that impact phenotype (22).

Chromatin accessibility can be a strong predictor of *cis*-regulatory functionality, as transcriptional regulators both affect chromatin conformation and themselves often require open chromatin to interact with their cognate *cis*-elements (23). There is a single stretch of the *AN* promoter that lies within a region of open chromatin as determined by prior ATAC sequencing (7) (Fig. 1*A*). Remarkably, this approximately 350 base pair region shows the highest density of CNSs within the AN promoter, containing three of the four dicot-level CNSs and an additional six Solanaceae-level CNSs (Fig. 1*B*). CRISPR editing of this region was highly efficient, perhaps due to this chromatin accessibility, and alleles perturbing this region showed strikingly strong phenotypes, with different alleles showing distinct effects on development. The multiple alleles generated allowed us to contrast the phenotypic effects of distinct small sequence perturbations in this CNS-enriched region. We proceeded with in-depth characterization of seven of these alleles, termed the *AN^Pro^* mutants (Fig. 1*B*).

### *AN* Promoter Hotspot Mutants Affect Inflorescence Architecture and Flower Development.

Wild-type tomato produces multiflowered inflorescences in a highly stereotyped “zig-zag” pattern of flower initiation (Fig. 1*C*), and both genetic and environmental variation can alter this distinctive architecture into more branched forms (22, 24, 25, 26). The largest deletions in the *AN^Pro^* region, *AN^Pro-1^* and *AN^Pro-2^*, include a 647 base pair deletion that removes the entirety of the conserved sequence and the majority of the region of open chromatin (*AN^Pro-1^*) and a 439 base pair guide-to-guide deletion that removes the entire region of conserved sequence (*AN^Pro-2^*). Deletions of regions that go beyond the guide site are a common occurrence when using multiguide constructs in genome editing and, in this case, gave us the advantage of comparing phenotypes between these two large deletions of this region. These alleles share a phenotype, proliferatively branching inflorescences that fail to form flowers (Fig. 1*C*). These inflorescences do infrequently form flower-like structures that have missing or unfused anther cones (Fig. 1 *C*, *Inset*), and some develop seed-bearing fruit (Fig. 1*C*), although the vast majority do not mature. These phenotypes are similar to a weak coding sequence allele of *AN* (20), indicating that the *AN^Pro-1^* and *AN^Pro-2^* mutants are hypomorphs that show partial loss of *AN* function, likely through the deletion of expression-promoting *cis*-regulatory sequence.

**Fig. 2.**
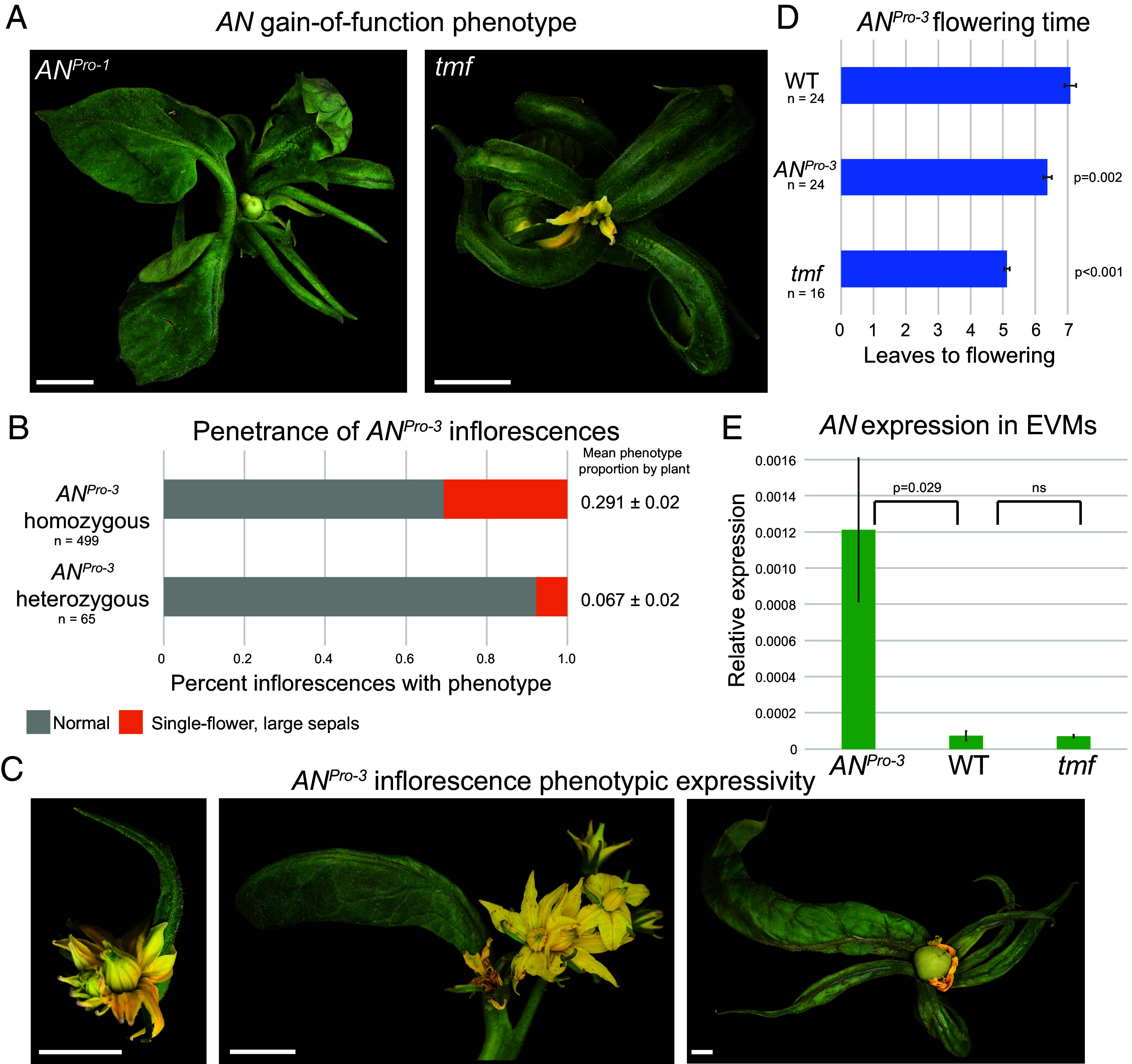
A specific allele of the *AN* promoter hotspot phenocopies gain-of-function *AN* mutants. (*A*) Representative inflorescences of *AN^Pro-1^* and *tmf* mutant inflorescences showing the gain-of-function phenotype. (Scale bar, 1 cm.) (*B*) Quantification of phenotypic frequency in *AN^Pro-3^* homozygous and heterozygous plants. The frequency of the single-flower phenotype per plant is given as a mean and SE. (*C*) Representative inflorescences of *AN^Pro-3^* mutants showing the gain-of-function phenotype. (Scale bar, 1 cm.) (*D*) Quantification of mean flowering time in WT, *AN^Pro-3^*, and *tmf* mutants. Error bars depict SE. Significant difference from WT was tested via Tukey’s HSD test. (*E*) Quantification of *AN* expression in WT, *AN^Pro-3^*, and *tmf* mutant inflorescences at the stage of early vegetative meristems. Significant difference from WT was tested via Tukey’s HSD test.

While both *AN^Pro-1^* and *AN^Pro-2^* homozygous mutant plants never form wild-type inflorescences, *AN^Pro-2^* plants show complete penetrance of the branched phenotype on all inflorescences, but *AN^Pro-1^* plants do not (Fig. 1*D*). Instead, while the majority of *AN^Pro-1^* inflorescences produce iterative branching and malformed flowers, occasional inflorescences on some plants show a striking contrasting phenotype—a single flower with abnormally large sepals (Fig. 2*A*). This phenotype is similar to the *AN* gain-of-function phenotype seen in transgenic plants where *AN* is expressed precociously under gene promoters that are activated in meristem maturation prior to the floral meristem (21). This phenotype is also the hallmark of *tmf* mutants (Fig. 2*A*), which show precocious expression of *AN* in transitional meristems (21). Unlike *tmf* mutants, which exhibit the gain-of-function phenotype on the first-formed inflorescence on the primary shoot, *AN^Pro-1^* plants show this phenotype stochastically throughout plant development.

While *AN^Pro-1^* plants infrequently exhibit this gain-of-function phenotype (Fig. 1*D*), it is much more penetrant in *AN^Pro-3^* mutants, with 30% of inflorescences from *AN^Pro-3^* homozygous mutant plants showing this phenotype (Fig. 2*B*), while other inflorescences on these plants showed wild-type architecture. *AN^Pro-3^* heterozygote plants also show the gain-of-function phenotype, though at a much-reduced frequency, suggesting this allele is dosage sensitive (Fig. 2*B*). Phenotypic expressivity is also variable between inflorescences (Fig. 2*C*). While all phenotypic inflorescences in both homozygous and heterozygous plants have at least one large leaf-like sepal, some have multiple, and the size of sepals varies between inflorescences. Furthermore, these inflorescences are frequently single flower or have fused flowers. These differences in penetrance and expressivity among plants and between inflorescences within individual plants indicate that *AN* function depends on highly sensitive temporal control to ensure robust inflorescence and floral development and shows that dosage of this critical developmental regulator can act as a tuning knob in development. This fact that these mutants phenocopy known mutants with aberrant *AN* expression further suggests that these *cis*-regulatory sequences control repression of *AN*, possibly to prevent precocious *AN* expression. In support, *AN^Pro-3^* plants flower on average of one leaf earlier than wild-type plants (Fig. 2*D*), and we found that unlike WT and *tmf* mutants, *AN* expression is already detectable in early vegetative meristems of *AN^Pro-3^* plants (Fig. 2*E*).

### *AN* Gain of Function Is Associated with Deletion of a Specific Transcription Factor Binding Site.

**Fig. 3.**
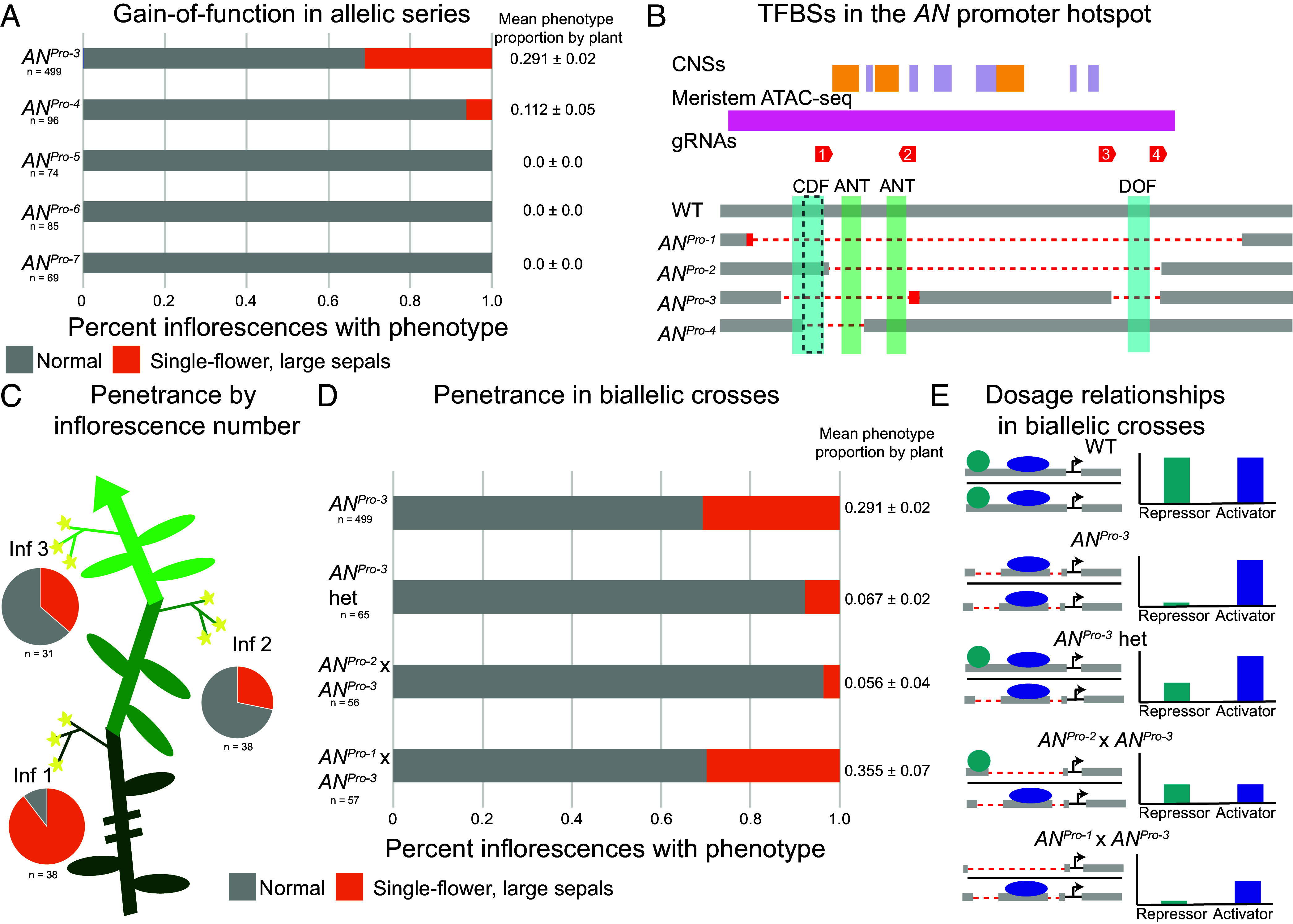
Penetrance and expressivity of the *AN* gain-of-function phenotype depend on genetic lesion, developmental stage, and genetic dosage. (*A*) Quantification of gain-of-function phenotype in *AN^Pro^* allelic series inflorescences. The frequency of the single-flower phenotype per plant is given as a mean and SE. (*B*) Depiction of the *AN^Pro^* hotspot showing putative transcription factor binding sites for DOF transcription factors, highlighted in blue. The 56 base pair region that is deleted in *AN^Pro-1^*, *AN^Pro-3^*, and *AN^Pro-4^*, but intact in *AN^Pro-2^*, is outlined by black hatch marks. (*C*) Quantification of the phenotypic frequency of *AN^Pro-3^* plants by inflorescence number. (*D*) Quantification of the phenotypic frequency of *AN^Pro^* biallelic crosses. The frequency of the single-flower phenotype per plant is given as a mean and SE. (*E*) Proposed mechanism by which biallelic mutants show sensitization or suppression of the *AN^Pro-3^* gain-of-function phenotype by balancing activity of activating and repressing trans-acting factors.

The *AN^Pro-3^* gain-of-function phenotype and early *AN* expression implies loss of repressor activity that allows precocious *AN* expression in early meristematic stages, potentially due to the elimination of repressive transcription factor binding sites (TFBSs). Given that the sequence perturbations in *AN^Pro-3^* are fairly large and complex, we isolated and characterized several smaller deletion alleles. While three alleles (*AN^Pro-5^_,_ AN^Pro-6^,* and *AN^Pro-7^*) as homozygous mutants (Fig. 1*B*) did not change inflorescence architecture (Fig. 3*A*), *AN^Pro-4^* homozygous mutants (Fig. 1*B*) displayed the gain-of-function phenotype (Fig. 3*A*). These alleles thus narrowed the region likely responsible for preventing early *AN* expression to a 56 bp sequence surrounding guide one that is completely deleted in *AN^Pro-1^_,_ AN^Pro-3^,* and *AN^Pro-4^* (Fig. 3*B*), all of which show *AN* gain of function to varying degrees of penetrance and expressivity. While other sequences deleted in these mutants must be important for penetrance (especially as the smallest deletion, *AN^Pro-4^*, shows the phenotype the least frequently), this particular region is the only shared deletion among all three alleles. Supporting how loss of this sequence underlies gain of function, these 56 base pairs are totally intact in *AN^Pro-7^* and only have one to five base pairs perturbed in *AN^Pro-5^* and *AN^Pro-6^,* all of which form wild-type inflorescences. Furthermore, *AN^Pro-2^*, which is a substantial genome perturbation which never shows the gain-of-function phenotype, also has this region entirely intact (Fig. 3*B*).

An analysis of TFBSs in the *AN^Pro^* sequence revealed putative binding sites for distinct transcription factor families. Several overlapping binding sites, immediately proximal to the first guide site itself, are for *CYCLING DOF FACTOR* (*CDF*) family transcription factors (Fig. 3*B*). *CDF*s repress precocious flowering in *Arabidopsis* through suppression of the flowering regulator *CONSTANS*, and *Arabidopsis cdf* mutants flower early in both long and short day conditions (27, 28). There are five *CDF* homologs in tomato, and previous results showed that expression of tomato *CDF*s under a constitutive promoter in *Arabidopsis* delays flowering (29), indicating that the ability of *CDF*s to repress flowering is conserved. Our results suggest that in tomato, *CDF*s repress flowering by blocking precocious *AN* expression during meristem maturation, aligning with our finding that *AN^Pro-3^* plants not only show aberrant inflorescence and floral development but also flower early relative to wild-type plants (Fig. 2*D*). While the *CDF* binding site is partially intact in *AN^Pro-4^*, it is completely removed in *AN^Pro-3^*, which may explain the difference in phenotypic penetrance between the two alleles. Additionally, there is a putative *DOF* transcription factor binding site deleted in *AN^Pro-3^* that is intact in *AN^Pro-4^* (Fig. 3*B*), which may serve as a redundant binding site for CDFs. Indeed, beyond the CDFs, other DOF transcription factors in tomato control inflorescence complexity (30), indicating a potential regulatory role also for DOFs in *AN* function during flower development.

The loss-of-function phenotype of *AN^Pro-1^* and *AN^Pro-2^* mutants likely is associated with a similar loss binding of trans-acting factors due to sequence deletion, though conversely to the gain-of-function mutants we expect to see deletion of activating TFBSs in these alleles. Analysis of the large region deleted in *AN^Pro-1^* and *AN^Pro−^* showed putative binding sites for multiple transcriptional activators, including for MADS-box (31–33) and MYB (34, 35) transcription factors. Intriguingly, two binding sites for the AP2 transcription factor AINTEGUMENTA (ANT) (36) were also identified in this region (Fig. 3*B*). *ANT* is a known regulator of *LEAFY*, *UFO*’s transcription factor coregulator (37), so this may be a mechanism by which expression of these two genes are coordinated in tomato, as *ANT* regulation of both *AN* and the *LEAFY* ortholog, *FALSIFLORA* (38), could promote flower formation. Alternatively, given that *AN* is only activated in the floral meristem in tomato, similar to the expression pattern of *LFY* in *Arabidopsis*, *AN* and *LFY* may be closer “expression orthologs” than their respective true evolutionary orthologs, due to *ANT* binding to the respective promoters in the two species.

### Penetrance and Expressivity of *AN* Gain of Function Depends on Developmental Stage and Dosage.

The variable penetrance and expressivity of the *AN* gain-of-function phenotype (Fig. 2 *B* and *C*) shows that there is stochasticity in which inflorescences manifest early *AN* expression or the degree to which this expression impacts phenotype. The penetrance of the gain-of-function phenotype depends on the order in which inflorescences develop on the plant, with phenotypic inflorescences developing earlier and later inflorescences more likely to show normal architecture (Fig. 3*C*). Phenotypic expressivity also varies by inflorescence number, with more severe phenotypes, such as single-flower inflorescences with multiple enlarged sepals, emerging more frequently on earlier inflorescences whereas multiflower inflorescences and flowers with a single enlarged sepal are more frequent on later developing inflorescences. This stochasticity suggests that activity of an *AN* repressor that binds to the deleted sequence can influence penetrance and expressivity, possibly by indirectly influencing the initial maturation states of subsequently formed axillary meristems (39). As tomato is a sympodial system where shoot meristems terminate in floral meristems and new specialized axillary (sympodial) shoots iteratively arise to continue growth, *AN*’s temporal expression patterns in a given transitioning floral meristem can potentially impact the development of the sympodial meristem developing at its base (21). Notably, *tmf* mutant plants also show the gain-of-function phenotype most frequently on the first inflorescence on a plant, with later axillary shoots developing normally (21). These observations are further reinforced by the partial expressivity of the *AN^Pro-3^* allele when heterozygous with an intact functional allele (Fig. 2*B*), reflecting a semidominant dosage relationship.

To further understand how dosage affects the expressivity of *AN^Pro-3^*, we generated biallelic mutant plants between *AN^Pro-3^* and our two hypomorphic loss-of-function alleles: *AN^Pro-1^* and *AN^Pro-^*^2^. We compared the gain-of-function expressivity in these plants to heterozygous *AN^Pro-3^* plants (Fig. 3*D*). *AN^Pro-2^* × *AN^Pro-3^* biallelic inflorescences exhibit the gain-of-function phenotype very rarely, at a lower proportion than *AN^Pro-3^* heterozygotes, likely because the combined dosage of a gain- and a loss-of-function allele suppresses this phenotype as balanced dosage is reestablished (Fig. 3*E*). Interestingly, *AN^Pro-1^* × *AN^Pro-3^* plants show the gain-of-function phenotype at a similar rate as *AN^Pro-3^* homozygotes (Fig. 3*D*), even though *AN^Pro-1^* homozygotes primarily show loss-of-function morphology (Fig. 1*D*). This may be because *AN^Pro-1^*, the largest deletion in this region, likely includes deletions for the binding of both transcriptional activators and repressors (Fig. 3*B*). The *AN^Pro-1^* × *AN^Pro-3^* genotype prevents binding for both activators and repressors at one allele (*AN^Pro-1^*), but for only repressors at the other (*AN^Pro-3^*), causing gain of function (Fig. 3*E*). These results suggest that balanced dosage of activator and repressor activity in the *AN* promoter is essential for stereotyped inflorescence architecture.

### Shared CNSs Between Tomato and *Arabidopsis* are Dispersed Upstream of *UFO* but Maintain Function.

**Fig. 4.**
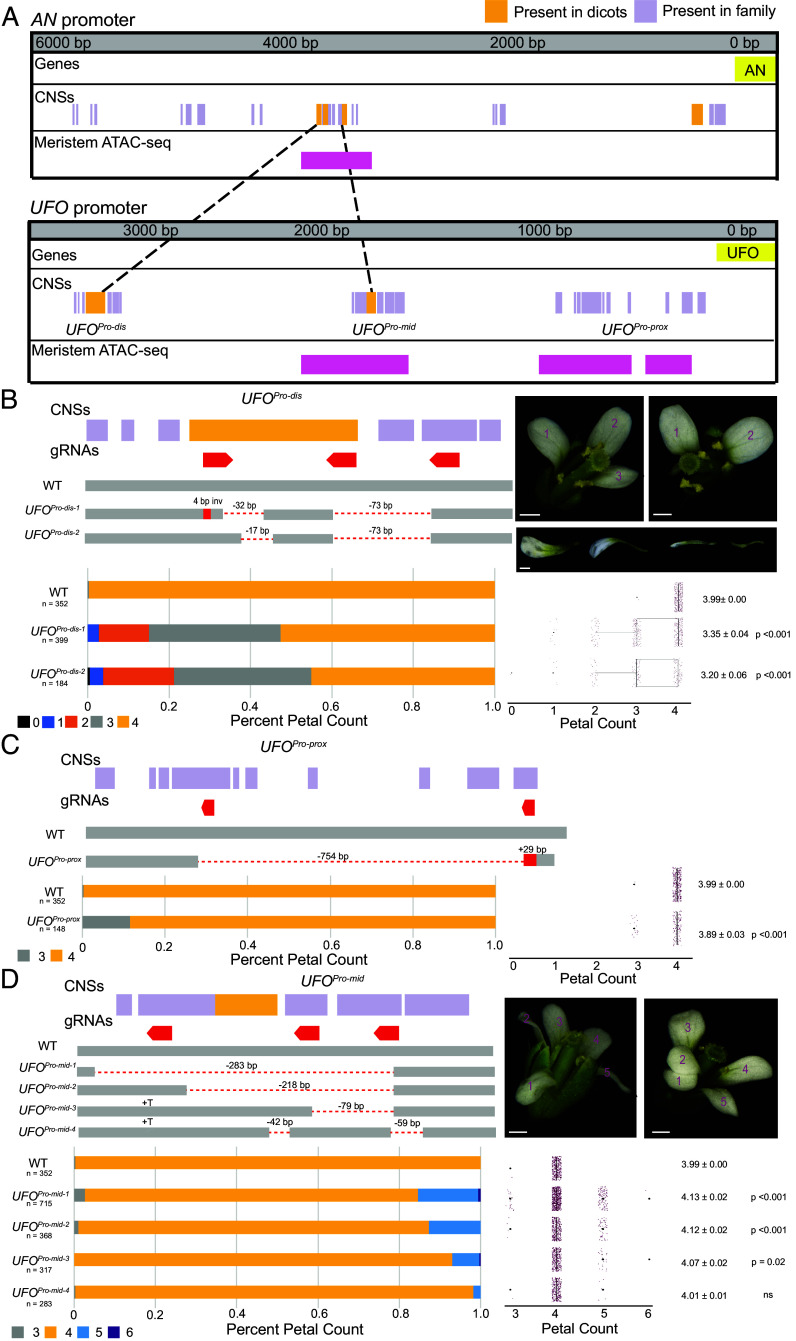
Perturbation of *UFO* CNSs in *Arabidopsis* breaks petal number canalization. (*A*) Depiction of CNSs and open chromatin in the *AN* and *UFO* promoters and sequence homology of dicot-wide CNSs between the two promoters. (*B*) *UFO^Pro-dis^* alleles are depicted and representative flower and petal images are shown. (Scale bar, 500 μm.) Petal counts are depicted as proportions in stacked bars and counts in boxplots. Average petal count and SE are shown. Significant difference from WT was tested via Tukey’s HSD test. (*C*) *UFO^Pro-prox^* allele is depicted. Petal counts are depicted as proportions in stacked bars and counts in boxplots. Average petal count and SE are shown. Significant difference from WT was tested via Tukey’s HSD test. (*D*) *UFO^Pro-mid^* alleles are depicted, and representative flower images are shown. (Scale bar, 500 μm.) Petal counts are depicted as proportions in stacked bars and counts in boxplots. Average petal count and SE are shown. Significant difference from WT was tested via Tukey’s HSD test.

The pronounced gain- and loss-of-function inflorescence phenotypes in the *AN^Pro^* allelic series suggest that a balance between activator and repressor activity in tightly temporally regulated developmental pathways ensures that core regulators function at precise required timepoints in diverse species. Using Conservatory (7), we found that three dicot-level CNSs share homology with regions of the *Arabidopsis UFO* promoter, spanning 140 million years of evolution. While the sequences are similar, the organization of these CNSs has changed substantially, as CNSs that are proximal in the tomato *AN* promoter are dispersed throughout the *Arabidopsis UFO* promoter (Fig. 4*A*). Two of the *AN* CNSs that Conservatory identified as shared among diverged dicot species map to a *UFO* CNS ~3 kbp upstream of the coding sequence, and the third maps to a CNS ~1.7 kbp upstream (Fig. 4*A*). We mutagenized *UFO* CNSs using CRISPR-Cas9 and generated multiple deletions in three regions, designated *UFO^Pro-dis^*, *UFO^Pro-mid^*, and *UFO^Pro-prox^*. Previously published ATAC sequencing of *ap1 cal* inflorescence meristem tissue (40) showed that two of these regions, *UFO^Pro-mid^* and *UFO^Pro-prox^*, are within open chromatin (Fig. 4*A*), similar to the *AN^Pro^* region of the *AN* promoter. In contrast to *AN^Pro^* mutants, none of the *UFO* mutants showed a loss of floral identity. However, all *UFO^Pro^* mutants that impacted CNSs affected petal development, specifically petal number. Importantly, a deletion in a 1 kbp region having no CNSs between the *UFO^Pro-dis^* and *UFO^Pro-mid^* alleles was indistinguishable from wild-type plants (*SI Appendix*, Fig. S2*A*), suggesting that these CNSs are strongly informative of *cis-*regulatory function.

In *Arabidopsis*, petal number is highly canalized at four petals per flower (41), and decanalization is rare, as most floral homeotic mutants lose petals entirely or aberrantly produce petals in other floral whorls (42). We found that different *UFO* promoter alleles disrupt robustness of petal number in opposite phenotypic directions. In *UFO^Pro-dis^* plants, approximately half of the flowers on a given individual plant form less than four petals (Fig. 4*B*), and this difference from the wild type was highly significant in both alleles. Strong *UFO* coding mutants lose all petals and stamens, but weaker alleles show variable petal number and petal homeotic conversion (12), suggesting that *UFO^Pro-dis^* mutants are hypomorphs. Interestingly, stamen number is intact in *UFO^Pro-dis^* mutants. This division of the pleiotropic roles of *UFO* caused by targeting distinct CNSs may be because specific CNSs control specific developmental processes (7), in this case petal initiation. Alternatively, the distinct processes that *UFO* regulates may be more or less sensitive to quantitative changes in *UFO* expression and function, with the petal whorl being most sensitive to changes to *UFO cis*-regulation during specification of floral meristem and organ identity. Supporting this difference in sensitivity are previously identified insertional mutants in the *UFO* promoter, which also affected petal but not stamen development (43). Interestingly, these mutants lie in a nonconserved region of the promoter downstream of *UFO^Pro-dis^*, suggesting it may be the displacement of the *UFO^Pro-dis^* CNS by large T-DNA insertions that drives loss of petals in these mutants.

Petal formation is also aberrant in *UFO^Pro-dis^* plants, with mutants showing often smaller and misshapen petals compared to wild-type plants (Fig. 4*B*) This suggests petal initiation is a dose-dependent development process with quantitative outputs—changing the dosage of expression and function of *UFO* can affect petal formation in regard to the shape and size of the petals, not just their number. The variable expressivity among different flowers of *UFO^Pro-dis^* mutant plants mirrors that of *AN^Pro^* mutant inflorescences, and the severity of the petal number decrease in *UFO^Pro-dis^* mutants increases as plants produce more flowers on inflorescence shoots, i.e. flowers that develop later on a given inflorescence have fewer petals (*SI Appendix*, Fig. S2*B*). Again, this is similar to the inflorescence age dependence of the severity of *AN^Pro-3^* mutants, suggesting that plant age and relative timepoints of inflorescence development can affect the degree to which flower development is able to buffer the impeded function of these CNSs in *UFO* expression.

We observed that *UFO^Pro-prox^* mutants show petal phenotypes similar to *UFO^Pro-dis^* mutants, though much less severe. These plants formed three-petaled flowers much less frequently, though petal number distribution was still significantly different than the wild type (Fig. 4*C*), suggesting that this CNS also plays a role in promoting petal development. Conversely, *UFO^Pro-mid^* mutants are decanalized in petal number in the opposite direction, increasing petal number to five, and also occasionally forming three-petaled flowers. The more severe *UFO^Pro-mid^* mutants correlated with larger deletions within this CNS, with *UFO^Pro-mid-1^*, *UFO^Pro-mid-2^*, and *UFO^Pro-mid-3^* all showing a petal distribution significantly different than the wild type (Fig. 4*D*). These differences between *UFO^Pro^* mutants, much like the opposing phenotypes of *AN^Pro^* mutants, suggest that both activator and repressor transcriptional machinery are binding at these CNSs, leading to precision in the temporal and spatial control of gene expression that promotes robust development. Analysis of TFBSs in the *UFO^Pro-mid^* region identified a putative CDF5 binding site, indicating that there may be shared *cis*–trans regulation of the gain-of-function *UFO* and *AN* phenotypes between *Arabidopsis* and tomato.

## Discussion

The question of how CNS organization impacts function is of interest to developmental biologists, and a combination of mutational approaches on native sequence (2, 18, 44) and systematic dissection of promoter architecture in synthetic systems (45) are complementary techniques toward a more comprehensive understanding of *cis-*regulatory grammar. Using targeted genome editing of homologous noncoding sequences across broad evolutionary distances, we found that in two highly diverged plant species, conserved sequence is highly predictive of *cis-*regulatory functionality during flowering. While *UFO* CNSs share homology between tomato and *Arabidopsis*, their spacing and positions relative to the gene body and to each other are different. This so-called “grammar” of CNS positioning clearly affects how these short, conserved sequences exert their regulatory control on their cognate genes (46), as CNSs that are deleted in gain-of-function mutants in tomato (*AN^Pro-3^*) instead show a loss-of-function phenotype when perturbed in *Arabidopsis* (*UFO^Pro-dis^*). Recent work comparing *Arabidopsis* with its close relative *Capsella rubella* found similar shifts in function of conserved *cis*-regulatory sequence during evolution, due to changes in genomic and developmental contexts (47). It is also undeniable that these allelic series do not encapsulate all of *AN* and *UFO cis*-regulation, as in both species only hypomorphic phenotypes were generated, rather than null mutant phenotypes. With advances in CRISPR-Cas with less stringent requirements for guide targets (48, 49), more precise targeting of CNS can be implemented in the future.

CNS organization is not the only distinction between the *AN* and *UFO* promoters. The phenotypes emerging from the CNS allelic series also differ between tomato and *Arabidopsis*. *UFO^Pro^* mutants show defects in petal number canalization whereas *AN^Pro^* mutants show defects in inflorescence architecture and flower formation. These different phenotypes reflect divergence in the pleiotropic roles of *UFO* during flower development. In Solanaceae species, *UFO* orthologs drive the transition to flowering (19), causing gain-of-function *AN^Pro^* mutants to promote precocious *AN* expression and to flower early. In contrast, in *Arabidopsis*, *UFO* is expressed broadly during meristematic development and *LFY* expression instead drives flower formation (19). This may explain why *UFO* CNS gain-of-function mutants do not affect flower formation—as *LFY* expression remains intact in *UFO^Pro^* mutants, flowering is undisrupted. Notably, the sequence that is deleted in all *AN^Pro^* gain-of-function mutants is not in a region of conservation, but rather immediately proximal to a dicot-level CNS. Whether CNS-proximal sequence may be a global repository to encode lineage-specific *cis-*regulatory function (such as *UFO* expression driving flowering in the Solanaceae) remains to be seen. Work in animal systems has shown that de novo enhancer formation is more likely to generate phenotypic novelty than changes in conserved enhancer sequences (50), suggesting that nonconserved sequence may more likely to promote developmental divergence across evolutionary time.

In both tomato and *Arabidopsis*, disrupting CNSs strongly affects overlapping developmental programs that are critical for reproduction, inflorescence architecture, and petal number. Petal number in *Arabidopsis* is normally a fixed trait, with near invariant formation of four-petaled flowers across ecotypes and environmental conditions (41). Interestingly, *Cardamine hirsuta*, a close relative of *Arabidopsis*, has naturally decanalized petal number that is affected both by genetic background (51) and environmental conditions (52). Given that *Arabidopsis UFO^Pro^* mutants can recapitulate this decanalization of petal count observed in Cardamine, it would be interesting to explore how modulating *UFO* expression and function during petal organogenesis can reveal phenotypic variation more broadly across evolution. Though inflorescence architecture and floral organ number are already variable phenotypes in tomato, in part due to domestication (24), ablation of CNSs in the *AN^Pro^* mutants causes even stronger effects on flowering and floral development, reinforcing that CNSs of essential developmental genes are regulatory hubs for canalized development. *Arabidopsis* and tomato also differ in their inflorescence organization—while *Arabidopsis* exhibits monopodial growth with inflorescences budding off an indeterminately growing shoot apical meristem, tomato has a sympodial growth habit, with each meristem terminating into a differentiated flower and new growth continuing from specialized (sympodial) axillary meristems (39). These differences in growth habit could contribute to the differences in phenotypic severity between the *UFO^Pro^* and *AN^Pro^* allelic series, suggesting that developmental trajectory differences across evolution affect the phenotypic consequences of modulating *UFO* function.

In both species, CNS-targeting mutants showed incomplete penetrance and variable expressivity both between plants and among inflorescences and flowers within a given individual. This variation suggests that the phenotypic manifestation of these perturbations depends on developmental progression, genetic dosage, and environmental conditions. The incomplete penetrance of *AN^Pro-3^* phenotype in biallelic plants in particular hints to the extraordinary dosage dependence of *AN* expression and function, as the combination of gain- and loss-of function mutations in biallelic mutant plants return to robust inflorescence development, likely due to a rebalancing between activator and repressor activity. These shifts in phenotypic penetrance due to allelic dosage is only visible because the gain-of-function phenotype is semidominant and thus present in *AN^Pro-3^* heterozygous plants.These opposing deviations from robustness in distinct alleles show that regulation of *AN* and *UFO* expression is clearly an inflection point in flower formation across species. CNSs are primed to integrate activator and repressor regimes, the slightest shift between which can cause strong effects on development.

An obvious but challenging next step would be to link the precise molecular consequences of noncoding alleles with their phenotypic penetrance and expressivity. Advances in in vivo reporter assays (53) and scRNA sequencing (54) could elucidate the temporal and spatial expression patterns of transcriptional regulators such as *UFO* in rare cells and developmentally transient tissue types such as maturing floral meristems and connect these expression patterns to the incomplete penetrance and variable expressivity among individual inflorescences and plants. Genomic methods to quantify transcription factor binding (55, 56) could bridge these analyses in *cis*-regulation to the trans-acting factors that bind to these sequences. For all these methods, allelic series, both engineered as in this work (2, 18, 44) as well as those derived from natural variation in the germplasm (57), are prime genetic resources to explore this link between quantitative expression changes in critical developmental regulators and the degree of penetrance and expressivity changes displayed by these *cis*-regulatory alleles (58). The more knowledge gained on these inflection points in other developmental programs and involving other core genes, the more we can understand the underlying molecular inducers of penetrance and expressivity. With current genomic and gene editing tools to mimic natural variation and go beyond it, we can form a more complete picture of how robustness is maintained through the opposing functions of activating and repressing transcriptional machinery (59), and how these *cis-*regulatory regimes can provide unique targets and opportunities for trait engineering.

## Materials and Methods

### Plant Material, Growth Conditions, and Phenotyping.

*Solanum lycopersicum* cv. M82 is the background cultivar for all WT and transformed tomato mutagenesis experiments. Tomato seeds were sown directly in 96-well flats for 4 wk before being either transplanted to pots and grown in greenhouse conditions or transplanted directly to fields at Uplands Farm at Cold Spring Harbor Laboratory in summer growth seasons. The greenhouse uses natural and supplemental artificial light (from high-pressure sodium bulbs ~250 μmol/m^2^) in long-day conditions (16 h light, 8 h dark) and is maintained at a temperature between 26 to 28 °C (day) and 18 to 20 °C (night), with relative humidity 40 to 60%. Field-grown plants were grown with drip irrigation and standard fertilizer regimes. For each unique genotype, inflorescence phenotypes were characterized for at least four inflorescences from at least ten plants. Inflorescence phenotypes were quantified from the first-developing inflorescences on the primary and secondary shoot. For flowering time quantification, plants were grown in greenhouse conditions until flowering. Leaf count before the first inflorescences was quantified for sixteen to twenty-four plants for each genotype. All raw data are described in Dataset S1.

*Arabidopsis thaliana* ecotype Col-0 is the background cultivar for all WT and transformed *Arabidopsis* mutagenesis experiments. *Arabidopsis* plants were germinated on ½ MS plates and transplanted to 48-well flats for growth. Plants were grown in growth chambers under long day conditions (16 h light, 8 h dark) at 22 °C and light intensity ~100 μmol/m^2^. For each unique genotype, petal number was quantified from at least ten flowers from twelve plants. Petal number was qualified for the first-developing flowers on the primary shoot. All raw data are described in Dataset S1.

### CRISPR-Cas9 Mutagenesis, Plant Transformation, and Selection of Mutant Alleles.

Transgenic tomato seedlings were generated via CRISPR-Cas9 mutagenesis as previously described (60). Guides were selected for proximity to CNSs and were designed using Geneious Prime (https://www.geneious.com). Guide RNAs, Cas9, and kanamycin selection genes were cloned into a binary vector via Golden Gate assembly (60, 61). This vector was then transformed into tomato via *Agrobacterium tumefaciens* mediated transformation in tissue culture (60). Transgenic plants were screened for mutations using PCR primers surrounding the gRNA target sites and Sanger sequenced to determine mutant identity. First- or second-generation transgenic plants were backcrossed to WT and Cas9-negative progeny were selected for phenotypic characterization.

Transgenic *Arabidopsis* were also generated via CRISPR-Cas9 mutagenesis using the Golden Gate assembly method to clone binary vectors containing the guide RNAs, Cas9, a kanamycin selection cassette, as well as a pFAST-R selection cassette used for seed coat color screening for transformants (62, 63). The *Arabidopsis* cassette used an intronized Cas9 previously demonstrated to increase editing efficiency (64). Cloning of this cassette is described in (44). *Arabidopsis* plants were transformed with binary vectors using *A. tumefaciens* floral dip (65). Transgenic seeds were selected using fluorescent microscopy and germinated on ½ MS plates before transferring to soil at seven days postgermination. Initial editing generations [T1 plants from T0 (dipped) parents] were subjected to a heat cycling regime shown to increase Cas9 editing activity (66). Growth chambers were set to shift between 37 °C for 30 h and 22 °C for 42 h for 10 d, before returning to normal long day conditions. T1 flower DNA was genotyped to identify plants that showed editing. Seeds from these plants were counterselected by fluorescence for the absence of Cas9 and screened in the next generation for mutant identity and zygosity. T3 homozygous plants were phenotyped. All gRNA and genotyping primer sequences are available in Dataset S2.

### *Cis*-Regulatory Sequence Conservation Analyses and TFBS Prediction.

CNSs were identified via Conservatory (7) and ATAC sequencing peaks were obtained from previous work on meristem chromatin accessibility in our lab. CNSs are listed in Dataset S3. Transcription factor binding sites were predicted within the conserved regions in the *AN* and *UFO* promoters using FIMO in the MEME suite (67). The TFBS position frequency matrices used were acquired from the JASPAR CORE PFMs of plant collection (68). A *P*-value cutoff of 0.00001 was used to predict TFBSs.

### RNA Extraction and Quantification of *AN* Expression.

Seeds of the relevant genotypes were germinated on wet filter paper at 28 °C in the dark and transplanted to soil in 96-well plastic flats and grown in greenhouse conditions once germinated. Meristems were harvested 5 to 7 d after transplant after microscopy confirmation of the early vegetative meristem (EVM) stage. Thirty meristems per replicate were harvested with three biological replicates per genotype. Meristems were immediately flash-frozen in liquid nitrogen upon harvest and total RNA was extracted using TRIzol Reagent (Invitrogen). Five hundred ng of RNA was used for complementary DNA synthesis with the SuperScript IV VILO Master Mix (Invitrogen). Quantitative PCR was performed with gene-specific primers using the iQ SYBR Green SuperMix (Bio-Rad) reaction system on the CFX96 Real-Time system (Bio-Rad). Primer sequences are available in Dataset S2.

## Supplementary Material

Appendix 01 (PDF)

Dataset S01 (XLSX)

Dataset S02 (XLSX)

Dataset S03 (XLSX)

## Data Availability

All study data are included in the article and/or supporting information.
