# Supplementary material for: Antagonizing cis-regulatory elements of a conserved flowering gene mediate developmental robustness
Source: Proc Natl Acad Sci U S A. 2025 Feb 18;122(8):e2421990122. doi: 10.1073/pnas.2421990122 (PMC11874208; doi:10.1073/pnas.2421990122)
Supplement: Supplementary file 1 — Appendix 01 (PDF) [file pnas.2421990122.sapp.pdf]

PNAS

# Supporting Information for

Antagonizing *cis*-regulatory elements of a conserved flowering gene mediate developmental robustness

**Authors:** Amy Lanctot[1,2], Anat Hendelman[1,2], Peter Udilovich[2], Gina M. Robitaille[1,2], Zachary B. Lippman[1,2*]

**Author Affiliations:**
[1] Howard Hughes Medical Institute, Cold Spring Harbor Laboratory, Cold Spring Harbor, NY, USA 11724
[2] Cold Spring Harbor Laboratory, Cold Spring Harbor, NY, USA 11724

*** Corresponding Author**
Zachary B. Lippman
1 Bungtown Rd
Cold Spring Harbor Laboratory, Cold Spring Harbor, NY, USA 11724
Telephone: 516-367-8897
Email: lippman@cshl.edu

**This PDF file includes:**

Figures S1 to S2

**Other supporting materials for this manuscript include the following:**

Datasets S1 to S3

# Figure S1

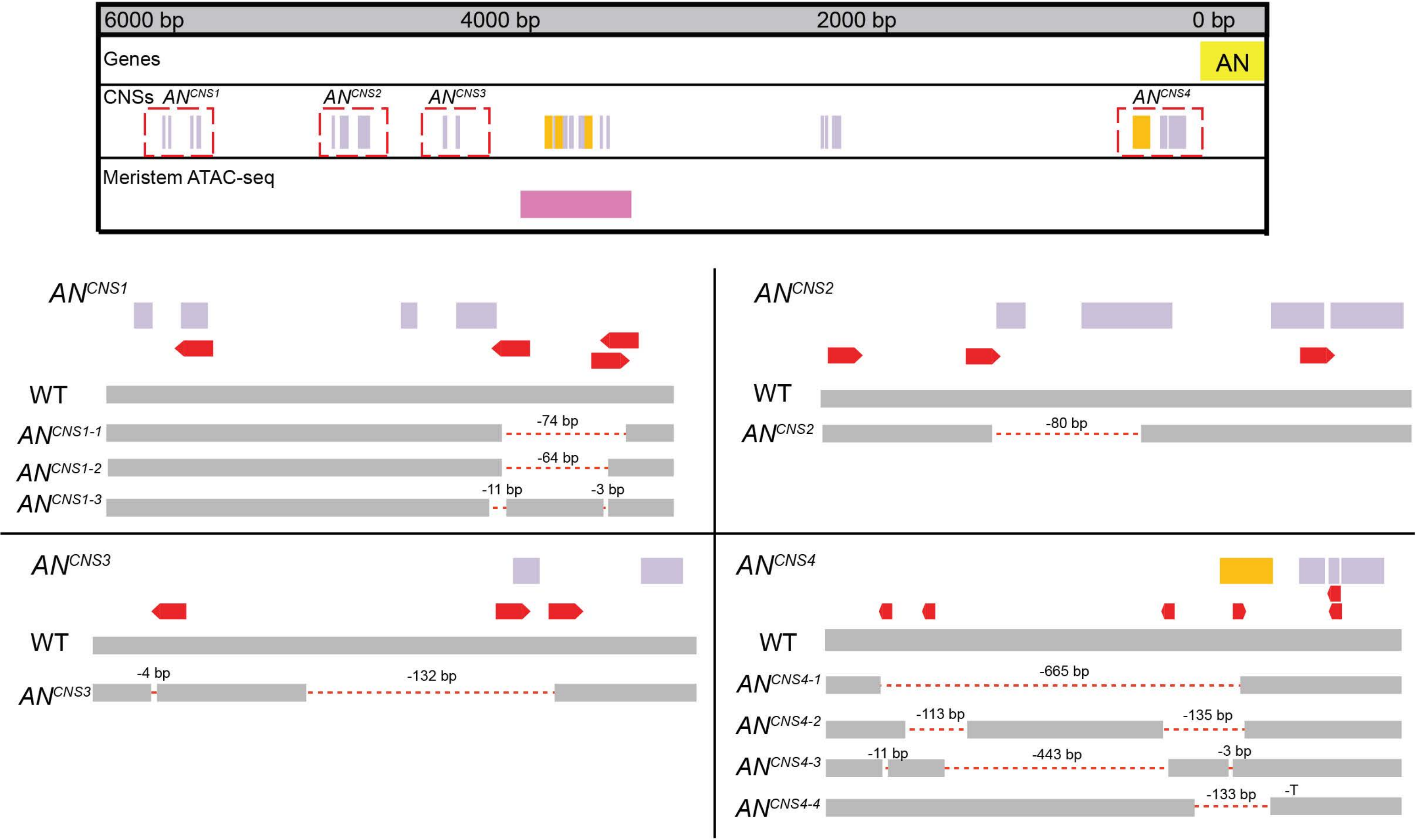


**Figure S1: CRISPR targeting of *AN* CNSs.** Depiction of CRISPR alleles generated in the four constructs targeting CNSs in the *AN* promoter that are not in regions of open chromatin.

# Figure S2

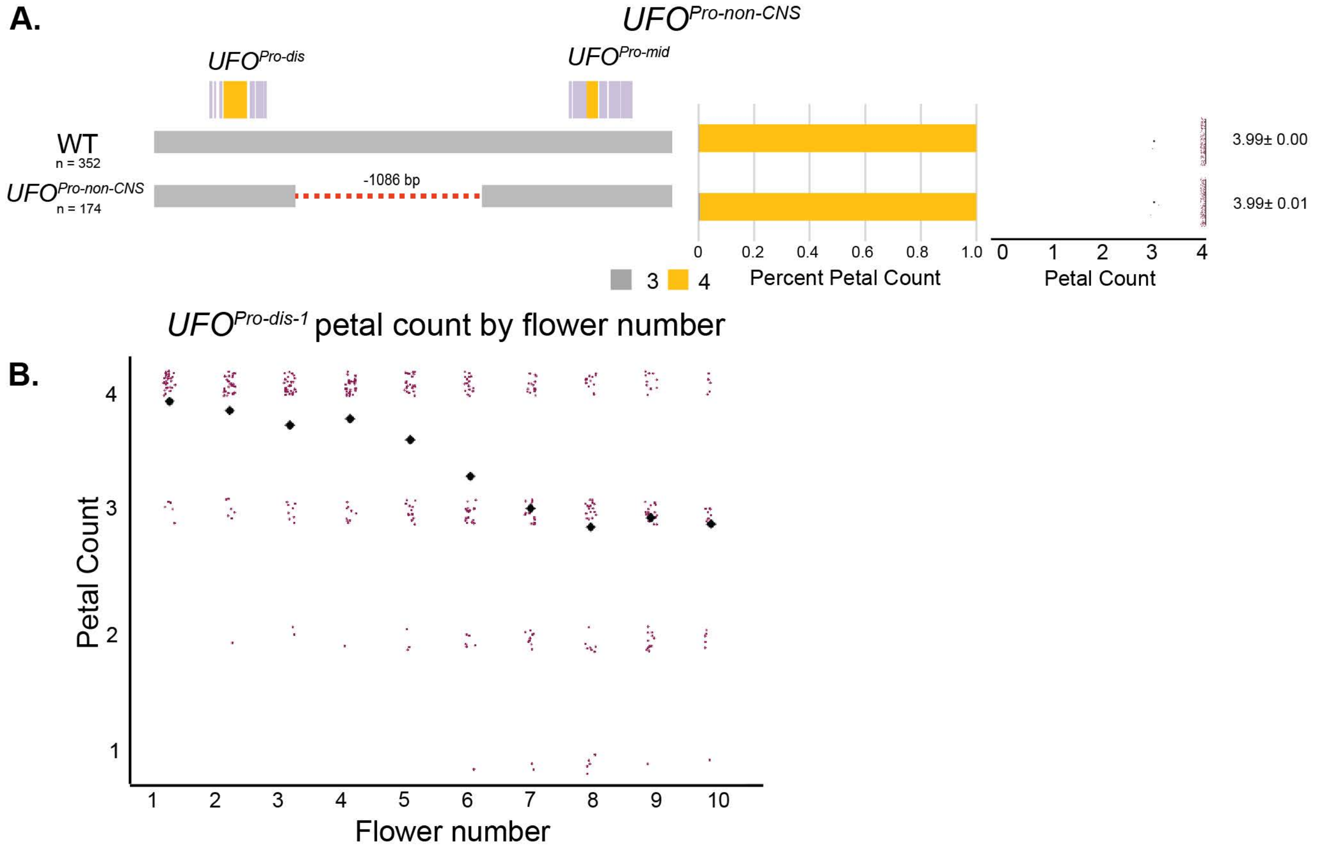


**Figure S2: Petal count decanalization depends on genetic lesion and flower number.** A. The *UFO*$^{Pro\text{-}non\text{-}CNS}$ allele is a ~1 kbp deletion between the distal and mid-promoter CNSs. *UFO*$^{Pro\text{-}non\text{-}CNS}$ mutants do not show petal number decanalization or any floral developmental defects. Petal counts are shown as proportions in stacked bars and total counts in boxplots. Average petal count and standard error shown. B. *UFO*$^{Pro\text{-}dis\text{-}1}$ mutants' petal count quantified by order of flower emergence. Petal counts are shown in magenta and mean petal counts by flower are depicted by black diamonds.

**Dataset S1 (separate file). Arabidopsis and tomato phenotyping raw data.** Raw counts of tomato inflorescence architecture, tomato flowering time, tomato gene expression, and Arabidopsis petal number.

**Dataset S2 (separate file). Oligos used in this study.** Sequences of guide RNAs, genotyping, and qPCR primers used in this study.

**Dataset S3 (separate file). CNSs identified by Conservatory in the *UFO* and *AN* promoters.** Names, sequences and identifiers of all CNSs identified by Conservatory in the promoters of the *UFO* and *AN* genes.
